# Neuromuscular exercise and pain neuroscience education compared with pain neuroscience education alone in patients with chronic pain after primary total knee arthroplasty: study protocol for the NEPNEP randomized controlled trial

**DOI:** 10.1186/s13063-020-4126-5

**Published:** 2020-02-24

**Authors:** Jesper Bie Larsen, Søren T. Skou, Lars Arendt-Nielsen, Ole Simonsen, Pascal Madeleine

**Affiliations:** 1 Translational Pain Biomarker, SMI, Department of Health Science and Technology, School of Medicine, Aalborg University, Aalborg, Denmark; 2 Sports Sciences – Performance and Technology, Department of Health Science and Technology, School of Medicine, Aalborg University, Aalborg, Denmark; 3 Research Unit for Musculoskeletal Function and Physiotherapy, Department of Sports Science and Clinical Biomechanics, University of Southern Denmark, Odense, Denmark; 4 Department of Physiotherapy and Occupational Therapy, Næstved-Slagelse-Ringsted Hospitals, Slagelse, Region Zealand Denmark; 5 Department of Orthopedic Surgery, Aalborg University Hospital, Aalborg, Denmark

**Keywords:** Total knee replacement, Chronic pain, Neuromuscular exercise, Pain neuroscience education

## Abstract

**Background:**

Total knee arthroplasty (TKA) is considered an effective treatment for pain relief and improved physical performances in end-stage knee osteoarthritis. However, several studies have reported less favorable outcomes after TKA with chronic pain rates of approximately 20%. Exercise might be an effective treatment strategy for chronic pain following TKA, but no randomized controlled trials have evaluated its effect. Therefore, the purpose of this randomized controlled trial is to investigate whether a 12-week neuromuscular exercise (NEuroMuscular EXercise training program for patients with knee or hip osteoarthritis assigned for total joint replacement; NEMEX-TJR) program combined with pain neuroscience education (PNE) provides greater pain relief and improvement in physical performances than PNE alone at 12 months follow-up in a population of patients with chronic pain after primary TKA.

**Methods:**

For this randomized controlled superiority trial, 120 patients with moderate-to-severe chronic pain after TKA are recruited from Aalborg University Hospital, Denmark. Patients are randomly assigned in a 1:1 ratio to one of two interventions: (a) NEMEX-TJR twice weekly for 12 weeks combined with two sessions of PNE or (b) two sessions of PNE given over 6 weeks. Assessment is performed at baseline before intervention and at 3, 6, and 12 months after initiation of the intervention. Outcome assessors are blinded toward group allocation. The primary outcome is the change in the Knee Injury and Osteoarthritis Outcome Score_4_ (KOOS_4_), defined as the mean score for the KOOS subscales pain, symptoms, activities of daily living, and quality of life. Secondary outcomes include all KOOS subscale scores and scores for PainDETECT, the Fear-Avoidance Beliefs Questionnaire, Global Perceived Effect, the Pain Catastrophizing Scale, pain intensities, temporal summation, conditioned pain modulation, and pressure pain thresholds. Physical performances are measured with walking, stair climbing, and chair standing tests as well as tests of muscle strength and power.

**Discussion:**

The findings will be useful in establishing effective treatment strategies for chronic pain after TKA. The randomized controlled trial involves rigorous scientific methods and uses clinically applicable interventions. The study interventions are conducted in clinical settings, thereby enhancing the possibility of future implementation of the treatments in the health care systems.

**Trial registration:**

ClinicalTrials.gov identifier: NCT03886259. Registered 22 March 2019. Ethics committee registration: N-20180046.

## Background

Osteoarthritis (OA) is considered the most frequent cause of disability and pain in the elderly population, and the knee joint is one of the joints most commonly affected [1, 2]. End-stage knee OA is often treated with a knee replacement. Total knee arthroplasty (TKA) is considered an effective treatment for pain relief and improved physical performance [3, 4]. However, less favorable outcomes such as chronic pain have been reported in approximately 20% of patients undergoing TKA [5]. Chronic pain in OA patients is believed to occur as a result of local pathological processes in and around the joint, genetic and metabolic factors and neuronal changes at several levels, including peripheral or central sensitization, reduced descending inhibition, and atrophy of cortical areas [6]. These mechanisms are considered important for the development of chronic pain after TKA [7, 8].

A systematic review reported that 20% of patients felt that TKA surgery had not been successful enough to resume their regular activities of daily living (ADL), including walking, stair climbing, dressing, and getting in and out of bed [9]. Therefore, the rehabilitation of patients with chronic pain after TKA needs to aim for both reducing pain and improving physical performance.

Guidelines for knee OA have established exercise as an effective treatment for both pain and functional impairment [10, 11]. For patients suffering from chronic pain after TKA, no such guidelines or standardized treatment regime exist. This is emphasized in two recent systematic reviews, summarizing the evidence regarding the treatment of chronic pain after TKA and reporting the absence of randomized controlled trials evaluating the effects of exercise therapy and other treatments [12, 13].

A neuromuscular exercise (NEuroMuscular EXercise training program for patients with knee or hip osteoarthritis assigned for total joint replacement; NEMEX-TJR) program developed by Ageberg et al. (2010) has been shown to be feasible and effective in treating pain and impaired function in patients with end-stage knee OA and immediately following TKA surgery [14, 15]. The NEMEX-TJR program aims at improving sensorimotor control and functional joint stability by using weight-bearing exercises with a focus on the quality and alignment of movements, optimal activation and loading of muscles, and postural control [14, 16]. Such a focus could also be relevant for patients with chronic pain after TKA. However, it is unknown whether the NEMEX-TJR program or any other exercise program is effective in a population of patients with chronic pain and impaired physical performance after TKA, highlighting the need for high-quality clinical trials.

Over the last few years, a shift in patient information from the biomedical model to a biopsychosocial model has been proposed as education for chronic pain patients [17, 18]. Since sensitization is a frequent phenomenon in chronic pain patients, a new approach to education is needed – one that targets pain neuroscience instead of focusing on the nociceptive pain [17]. Pain neuroscience education (PNE) has the potential to decrease anxiety and fear of movement after surgery and could therefore be useful for patients experiencing chronic pain after TKA [19]. Pain neuroscience education seems to provide the best results when delivered in conjunction with exercises, as these two treatments modalities might optimize each other [20].

We initiated the NEPNEP (Neuromuscular Exercises and Pain Neuroscience Education for chronic Pain) trial with the purpose of evaluating whether a 12-week NEMEX-TJR program combined with PNE provides greater pain relief and improvement in physical performances than PNE alone in a population of patients with chronic pain after primary TKA. We hypothesize that NEMEX-TJR and PNE in combination can provide greater pain relief and better physical performance compared to PNE alone after 12 months.

## Methods/design

### Study design

This study is a randomized controlled superiority trial with blinding of both the outcome assessor and the statistician analyzing the data. This study protocol follows the recommendations of the SPIRIT (Standard Protocol Items: Recommendations for Interventional Trials) guideline [21] (see supplementary file 1). In conjunction with the SPIRIT guideline, specific interventions were described based on the TIDieR (Template for Intervention Description and Replication) checklist [22] and the CERT (Consensus on Exercise Reporting Template) checklist [23] (see supplementary file 2).

### Ethical considerations

The trial is being conducted in accordance with the Helsinki Declaration, has been approved by the local ethics committee (the North Denmark Region Committee on Health Research Ethics, Aalborg, N-20180046), and is registered at ClinicalTrials.gov (NCT03886259; https://clinicaltrials.gov/ct2/show/NCT03886259). Any changes to the trial protocol will be communicated to the ethics committee and the clinical trial registry. All patients will be thoroughly informed about the trial before inclusion and will sign informed consent forms before participation. The NEMEX-TJR program used in the current trial has been found to be feasible and safe to use [14]. No adverse events, besides possible temporary soreness after the NEMEX-TJR program, are expected and therefore, no data monitoring committee has been established. If any serious adverse events occur during the trial, these will be reported to the North Denmark Region Committee on Health Research Ethics, Aalborg as soon as the principal investigator has been alerted.

### Participants and settings

**Fig. 1:**
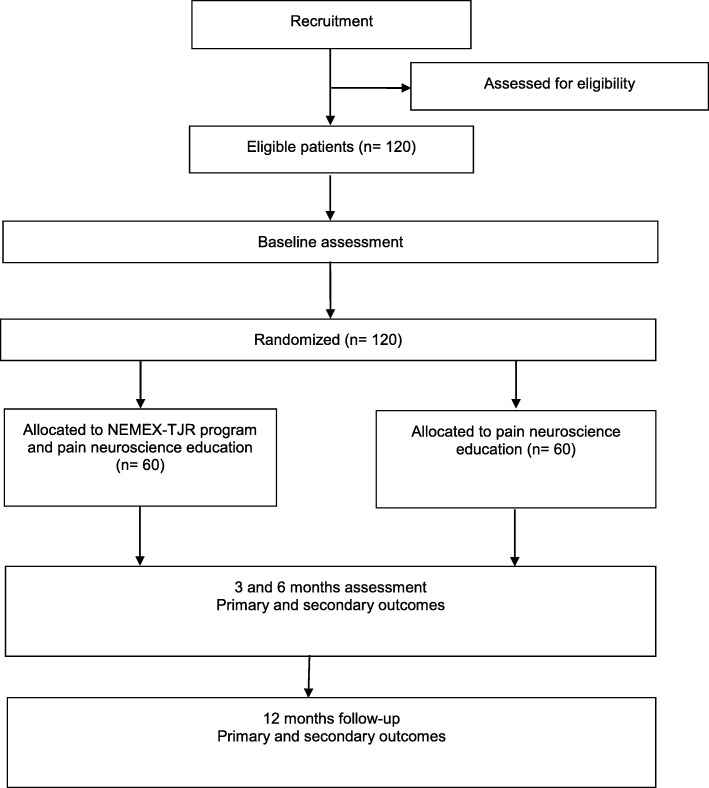
Flow diagram

A total of 120 patients will be recruited from the orthopedic surgical department at Aalborg University Hospital. Patients will be recruited from the hospital's patient research database and from screening patients referred to the involved orthopedic departments. To facilitate patient enrollment it will be possible to participate at one of the three local Departments of Occupational and Physiotherapy from the northern region of Denmark, thereby covering a larger geographical area. Patients will have their travel expenses reimbursed to avoid social inequality and increase adherence. Patients will be randomly assigned in a 1:1 ratio to undergo either 12 weeks of NEMEX-TJR combined with PNE or PNE alone. A flow diagram is presented in Fig. 1.

Patients will be eligible for inclusion if they meet the following criteria:

- male or female aged 40 to 80 years;
- body mass index (BMI) between 19 and 40 kg/m^2^;
- primary TKA due to OA ≥ 12 months postoperatively;
- for the index knee, a duration of knee pain > 6 months; and
- for the index knee, an average daily pain score ≥ 4 (moderate-to-severe pain) on a numeric rating scale (NRS; 0 (no pain) to 10 (maximum pain)) over the last week prior to recruitment.

Patients having the following attributes will be excluded from the trial:

- chronic pain due to loosening of implant or prosthesis failure requiring revision surgery;
- secondary causes of arthritis to the knee, such as rheumatoid arthritis or sequelae from previous accidents;
- surgery (including arthroscopy) of the index knee within 3 months prior to recruitment;
- injury to the index knee within 12 months prior to visit;
- acute pain, other than in the index knee, affecting the lower limb and/or trunk at the time of baseline testing;
- participation in other pain trials 2 weeks prior to recruitment;
- pregnancy;
- drug and alcohol abuse;
- rheumatoid arthritis, neurologic illnesses or primary pain area other than knee (e.g. low back pain or upper extremity pain); or
- lack of ability to adhere to protocol.

### Sample size

Based on the literature, a minimal clinically important difference of 10 points in the Knee injury and Osteoarthritis Outcome Score (KOOS) is commonly used [24]. The sample size calculation was based on the KOOS_4_ and defined as the mean score for the KOOS subscales of pain, symptoms, ADL, and quality of life. A sample size calculation was conducted to obtain a study power of 90% to detect a minimum improvement of 10 points on the KOOS_4_ in the NEMEX-TJR and PNE group compared with the PNE group (with a standard deviation [SD] of 15) [15, 25]. A two-sided significance level at 0.05 was applied. The calculation revealed that 49 patients will be required in each group. To account for possible missing data and a 20% loss from patients missing follow-ups, a total of 60 patients will be included in each group.

### Randomization and allocation concealment

The patients will be randomized in a 1:1 ratio to one of two treatment arms: (1) NEMEX-TJR and PNE or (2) PNE alone. Using computer-generated random numbers in permuted blocks of four to eight patients, the project manager will randomly assign patients to either of the treatment arms after the informed consent has been signed and the baseline assessment conducted.

### Interventions

Patients will participate in the interventions at the local Departments of Occupational and Physiotherapy in the northern region of Denmark. These departments are part of the Aalborg University Hospital, Denmark.

Patients in the exercise and PNE group will receive a 12-week rehabilitation program consisting of the NEMEX-TJR program [14]. The NEMEX-TJR program will be conducted as a 1-hour, group-based session twice a week for 12 weeks (24 sessions in all). Physiotherapists specifically trained to conduct the intervention will instruct and supervise the patients during the neuromuscular exercises, including individualization of the load and exercise difficulty based on each patient’s physical ability and pain intensity. Exercises will be initiated by a warm-up session consisting of 10 minutes of ergometer cycling at a self-selected intensity, followed by a circuit program consisting of exercises such as pelvic lifts, sit-ups, sliding exercises, lunges, rubber band exercises, chair stands, and stair climbing, with walking as a cool-down exercise. Exercises will be performed in two to three sets of 10 to 15 repetitions, with a short rest between each set and exercise. Exercises will be initiated at level 1 and could progress to levels 2 and 3. Progression (e.g. increasing the load or range of movement or changing the support surface) will be applied if the exercises are performed with high-quality and sensorimotor control based on visual inspection by the physiotherapist and if patients perceive the exercises as requiring minimal effort [14]. Pain intensity during and after training will be monitored. Due to the chronicity of the patients’ pain, a time-contingent approach to the exercises is preferred over a symptom-contingent method [26]. If a major flare-up in pain levels is experienced, the intensity and volume of training will be reduced until symptoms are “as usual” [14].

Both treatments groups will receive the same PNE consisting of two group-based educational sessions: one at the start of the trial period and one 6 weeks after the initiation of the trial. Both sessions will take 1 hour and be conducted by a physiotherapist trained in PNE. The sessions cover topics concerning the multifactorial nature of chronic pain, sensitization, hyperalgesia, allodynia, and plasticity of the brain, aiming at giving patients a better understanding of their chronic pain and thereby engaging the patients in the treatment. Information leaflets summing up the PNE topics will be handed out to the patients after the PNE sessions. During the sessions, it will be possible to ask questions and share experiences within the patient group.

### Assessments and blinding

**Fig. 2:**
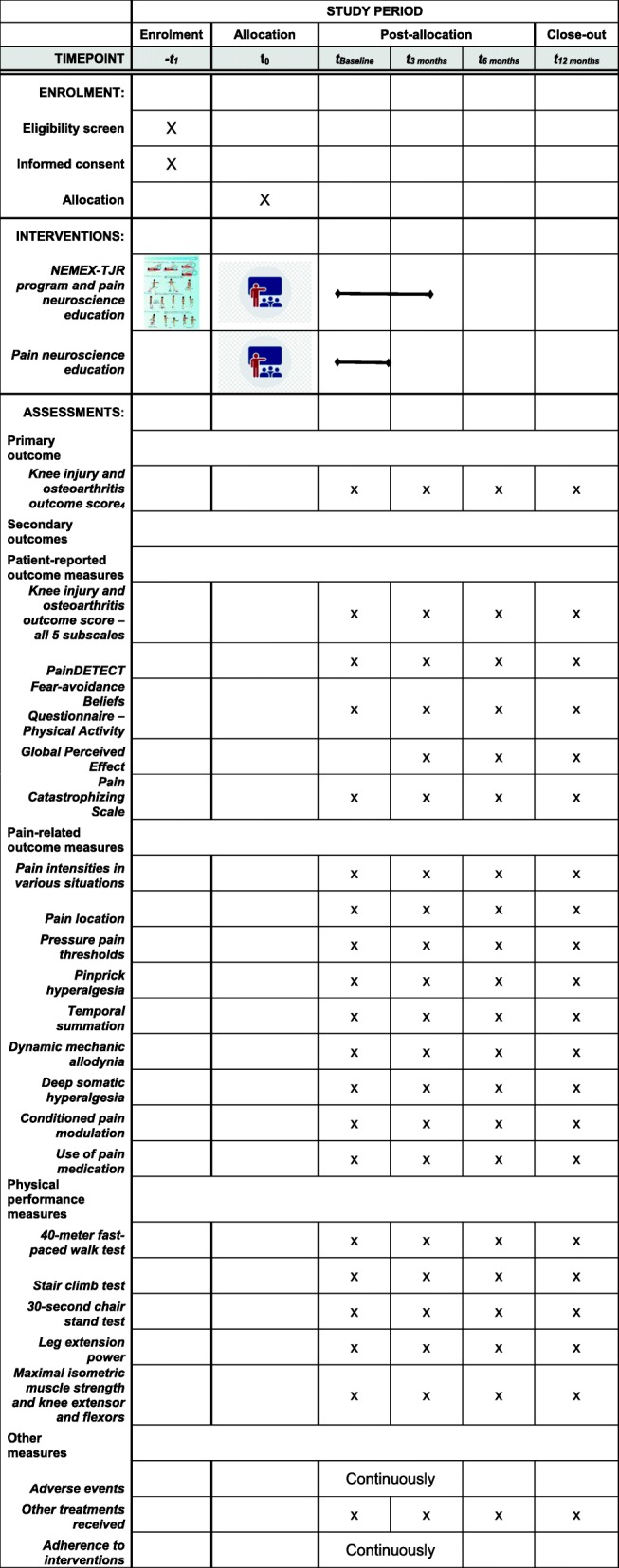
SPIRIT figure showing schedule of enrolment, interventions, and trial outcomes to be assessed

Baseline measurements will be completed prior to randomization and follow-up measurements will be conducted at 3, 6, and 12 months after initiation of the intervention. All assessment will take place at the Department of Occupational and Physiotherapy, Aalborg University Hospital, Denmark (see Fig. 2). Trained outcome assessors, blinded toward treatment allocation, will perform all assessments. Before the follow-up assessments, patients will be asked not to mention which treatment group they have been assigned to in order to maintain blinding. Demographic measures include gender, age, height, body mass, BMI, index knee, dominant leg, time since surgery, comorbidities, and scores on the Hospital Anxiety and Depression Scale [27].

### Outcomes

#### Primary outcome

##### Knee Injury and Osteoarthritis Outcome Score_4_ (KOOS_4_)

The primary outcome will be the change from baseline to 12 months in KOOS_4_, which will be an average of the four subscales of pain, symptoms, ADL, and quality of life from the patient-reported outcome measure KOOS. The KOOS_4_ ranges from 0 (worst) to 100 (best) [24]. A minimum change of 10 points is considered clinically relevant [25].

#### Secondary outcomes

##### Patient-reported outcome measures

Several patient-reported outcome measures will be collected (Fig. 2). The scores on all five KOOS subscales, including the sport-recreation subscale, will be included to support the clinical interpretation of the primary outcome [28]. The PainDETECT questionnaire is a screening tool that predicts the likelihood of a neuropathic pain component in chronic pain disorders [29]. The Fear-Avoidance Beliefs Questionnaire – Physical Activity is a four-item questionnaire for which a high score indicates a high degree of fear-avoidance beliefs [30, 31]. Global perceived effect will be assessed using the question: “How are your knee problems now compared with before you entered this study?” The question will be answered on a 7-point Likert scale ranging from “improved, an important improvement” to “worse, an important worsening” [32]. The Pain Catastrophizing Scale is a 13-item questionnaire developed to explore how catastrophizing affects pain experiences [33].

##### Pain-related outcome measures

Pain intensity ratings will be measured using NRS for the average daily pain intensity over the last week prior to the visit and the maximal pain intensity during rest (day and night), walking, and stair climbing. Patients will also be asked to draw their habitual pain distribution on anatomical body maps [34]. A handheld algometer (Somedic, Hörby, Sweden) with a 1-cm^2^ probe will be used to record the pressure pain threshold (PPT) in kPa, which can be reliably assessed using pressure algometry [35, 36]. The pressure will be increased gradually at a rate of 30 kPa/s until the pain threshold is reached and the patient presses a stop button. The PPTs will be measured three times for each point with an interval of a minimum of 20 seconds between each PPT assessment. The average of the three PPTs will be calculated for further analysis. Bedside tests for sensitization consist of pinprick hyperalgesia, temporal summation, dynamic mechanic allodynia, and deep somatic hyperalgesia. A single pinprick with a nylon filament (0.7 mm, Chicago Medical Supply) will be applied perpendicularly to the skin (until slight bending of the filament occurs, when a force of 75 gram is applied) and the patients rated the pain intensity on an NRS. Temporal summation will be measured using the nylon filament (0.7 mm) applied 10 times in an area of 1 cm^2^ with a frequency of 1/s. Then, the patients will rate the intensity of the last stimulus on an NRS. Dynamic mechanical allodynia will be examined using a cotton swab stroked four times on the skin (twice from each direction of a cross with 90° angles). The length of each stroke will be 3–5 cm. The patients will rate the pain intensity induced by the stroke on an NRS. Deep somatic hyperalgesia will be measured using a bedside pressure algometer (syringe). The air in the syringe will be compressed at a constant speed (1 ml/s) until the pressure becomes painful. The patients will be asked to indicate when the pressure became painful (threshold in ml). Pressure pain thresholds and bedside tests for sensitization will be recorded in the area of the index knee, adjacent to the knee (10 cm above the knee, ventral thigh), and extra-segmentally on the medial side of the forearm (muscle belly of the flexor digitorum superficialis). Conditioned pain modulation will be assessed using PPT as test stimuli and a spring-based pressure clamp applying a force of 1.3 kg as conditioning stimuli. Test stimuli will be applied to the middle part of the tibialis anterior muscle of the non-affected side and conditioning stimuli will be applied to the ipsilateral earlobe. The patients will be asked to report their use of pain medication during last week (yes/no), including the number of paracetamols (1 g), ibuprofen (400 mg), and other nonsteroidal anti-inflammatory drugs.

##### Physical performance measures

Five objective physical performance measures will be collected (Fig. 2). Based on the Osteoarthritis Research Society Internationals core recommendations for physical performance measures, a 40-meter fast-paced walk test, a stair climb test, and a 30-second chair stand test will be performed [37]. Leg extension power (Nottingham Power Rig, Nottingham, UK) expressed as the product of force and velocity in a single-leg simultaneous hip and knee extension will be measured. The force will be recorded for each push (with 30 seconds rest between trials) until the patients reach a plateau, defined as two successive measurements below the highest measurement. A minimum of six trials to minimize the learning effect and a maximum of 12 trials to minimize fatigue will be conducted and peak measurements in Watts obtained, which is a methodology used frequently and illustrating excellent reliability [38–40]. Maximal voluntary force from the knee extensors and flexors will be measured bilaterally in isometric conditions with a handheld dynamometer (Lafayette Manual Muscle Tester, Loughborough, UK or MicroFET2, Hoggan Scientific, LLC, Salt Lake City UT, USA). The patients will be asked to exert a maximum voluntary isometric contraction lasting 5 seconds against the hand-held dynamometer. Three trials of each test will be performed and the peak value in Newtons will be the outcome score, which is an approach that has illustrated excellent test-retest reliability [41]. A 30-second break between each measurement will be given.

##### Other outcomes

Adverse events that may occurr during the trial period will be identified by the patients (self-reported) and by the physiotherapists supervising the interventions (observations). Adverse events are characterized as occurring in the index knee or sites other than the index knee and serious events are defined according to the definitions from the U.S. Food and Drug Administration [42]. Non-serious adverse events are comprised of all other occurring events. Participants are allowed to receive other types of treatment at their own discretion. The other types of treatment received during the trial period will be self-reported. Other treatment is defined as treatments that the patient had initiated because of the index knee such as acupuncture, manual therapy, and surgery. Adherence to interventions will be registered for both groups. In the NEMEX-TJR and PNE group, the number of attended exercise sessions and PNE sessions will be recorded (i.e. number of sessions out of 24 exercises sessions and two PNE sessions). In the PNE group, the number of PNE sessions will be recorded (i.e. number of sessions out of two possible sessions).

### Statistics

The statistical analysis of the primary outcome will be performed according to an intention-to-treat principle. Statistical tests will be dependent of data distribution. We expect data to be normally distributed, and therefore, aim at using a repeated measures mixed model with patients as random effect and time (baseline and 3, 6, and 12 months) and treatment arm (NEMEX-TJR and PNE or PNE alone) as fixed effects, and with adjustments for baseline imbalance. No imputation will take place. Secondary outcomes and other endpoints will be analyzed similarly to the primary outcome. The frequency of adverse events will be compared between groups at the 12-month follow-up using a Poisson regression model with robust error variance. Categorical outcomes will be analyzed using a Χ^2^ test, Fisher exact test, or a Mann-Whitney *U* test as appropriate. A per-protocol analysis will be performed for the primary outcome, excluding patients who had poor adherence to the intervention, defined as participating in less than 75% of the exercise sessions and not attending both PNE sessions, and excluding patients who undergo additional surgery during follow-up. A 95% confidence interval (CI) excluding 10 points or more in the KOOS_4_ score will be interpreted as a lack of a clinically meaningful difference between groups. *P* values and 95% CIs will be presented. All authors will have access to the final anonymized trial dataset.

### Trial steering committee

See title page for members of the trial steering committee. All members participated in the conception of the study design and procured funding. The principal investigator (JBL) is coordinating the ongoing trial. The trial steering committee reviews the progress of the trial and agrees the necessary changes in the protocol, if any.

### Data collection and management

All obtained results will be collected using a test score protocol or fulfilling questionnaires and thereafter, entered into Excel (version 2016, Microsoft Corporation, Redmond, WA, USA). From Excel, data will be transferred into SPSS version 25.0 (SPSS Inc., Chicago, IL, USA) for statistical analyses. All collected test score protocols and questionnaires will be kept in a locked place as back up. Anonymized study data in electronically format will be stored at an encrypted network. Access to study data is restricted and cannot be accessed without permission.

### Audit

The North Denmark Region Committee on Health Research Ethics selects a number of approved studies for audit every year. These audits are independent from the trial steering committee and possible study sponsors.

### Publication

Results will be published regardless of outcome. Authorship will be determined based on the guidelines from the International Committee of Medical Journal Editors. The authors do not have any publication restrictions.

## Discussion

The aim of this trial is to evaluate the effects of a treatment that could potentially relieve pain and improve physical performance as well as provide education to assist patients in handling their chronic pain and disability. This is of definite importance since no other randomized controlled trial has been conducted regarding exercise as treatment for chronic pain after TKA [12, 13]. The trial builds on the presence of sensitization in several studies examining painful knee OA and chronic pain after TKA [7, 43–46] and evaluates if it is possible to modulate sensitization outcomes by using NEMEX-TJR in combination with PNE.

The strengths of the trial are the use of rigorous methods that include randomized allocation, blinding of outcome assessors, and the use of clinical applicable interventions. The trial interventions are conducted in clinical settings, thereby enhancing potential future implementation of the treatments in health care systems.

There are some potential limitations of the trial. Since the patients have chronic pain, some may use pain medication. This could interfere with the assessment of pain. In order to reduce possible bias from this, the use and dosage of medication will be assessed, allowing the study group to evaluate whether the use of pain medication increased or declined during the trial. Chronic pain patients often have comorbidities or other painful musculoskeletal disorders [47] that may influence how they respond to the interventions. One way to avoid this influence would be to sample a population of patients with no other pain sites or comorbidities besides knee pain following TKA. Such a homogeneous group would not reflect real-life patients and therefore, the results would not be generalizable. Due to their chronic pain and disabilities, patients might decide to seek help or other treatments during the trial. Patients who receive PNE alone might be more interested in seeking other treatments than the exercise group, which will already be receiving additional treatment twice a week. If this occurs, it might counterbalance the possible differences between the groups. To avoid this bias, we will collect data on other treatments received during the trial period, which will allow us to evaluate whether this parameter might have had an impact. Since the group receiving NEMEX-TJR in combination with PNE has 24 more sessions with the physiotherapist, there is an inherent risk that part of the explanation for a possible larger effect in this group will be due to the increased attention that they receive alongside a stronger patient-practitioner interaction [48]. Large contributions from contextual factors have been observed in treatment effects, although not thoroughly studied in trials of exercise and education as treatment of knee pain [49]. Finally, no process or economic evaluation will be conducted within the present trial. Such evaluations will be important to ease and justify a more general implementation of the NEPNEP intervention in TKA patients suffering from chronic pain.

### Trial status

The trial is ongoing and is currently recruiting patients. Recruitment was initiated on April 12, 2019 and is expected to be completed by the end of December 2021. This protocol is based on protocol version 4.0 of the 16 August 2018. The trial was registered with ClinicalTrials.gov (NCT03886259) on the 22 March 2019.

## Supplementary information

**Additional file 1.** SPIRIT 2013 Checklist for the NEPNEP trial.

**Additional file 2.** The TIDieR (Template for Intervention Description and Replication) Checklist for the NEPNEP trial.

## Data Availability

The data sets used and analyzed during the trial will be available from the principal investigator on reasonable request.

## Abbreviations

ADL: Activities of daily living
BMI: Body mass index
CERT: Consensus on Exercise Reporting Template
CI: Confidence interval
KOOS: Knee injury and Osteoarthritis Outcome Score
NEMEX-TJR: NEuroMuscular EXercise training program for patients with knee or hip osteoarthritis assigned for total joint replacement
NRS: Numerical rating scale
OA: Osteoarthritis
PNE: Pain neuroscience education
PPT: Pressure pain threshold
SD: Standard deviation
SPIRIT: Standard Protocol Items: Recommendations for Interventional Trials
TIDieR: Template for Intervention Description and Replication
TKA: Total knee arthroplasty
